# Effects of electron acceptors on sulphate reduction activity in activated sludge processes

**DOI:** 10.1007/s00253-017-8340-3

**Published:** 2017-05-25

**Authors:** Francisco Rubio-Rincón, Carlos Lopez-Vazquez, Laurens Welles, Tessa van den Brand, Ben Abbas, Mark van Loosdrecht, Damir Brdjanovic

**Affiliations:** 1 Sanitary Engineering Chair Group, Department of Environmental Engineering and Water Technology, UNESCO-IHE Institute for Water Education, Westvest 7, 2611AX Delft, The Netherlands; 2 Department of Biotechnology, Delft University of Technology, Van der Maasweg 9, 2629 HZ Delft, The Netherlands; 3 KWR Watercycle Research Institute, Groningenhaven 7, 3433 PE Nieuwegein, The Netherlands

**Keywords:** Sulphate reduction activity, Sulphate-reducing bacteria, Biological nutrients removal, Wastewater treatment, Electron acceptor inhibition

## Abstract

The concentration of sulphate present in wastewater can vary from 10 to 500 mg SO_4_^2−^/L. During anaerobic conditions, sulphate is reduced to sulphide by sulphate-reducing bacteria (SRB). Sulphide generation is undesired in wastewater treatment plants (WWTPs). Previous research indicated that SRB are inhibited by the presence of electron acceptors (such as O_2_, NO_3_ and NO_2_). However, the contact times and concentrations used in those studies are by far higher than occur in WWTPs. Since sulphide can influence the biological nitrogen and phosphorus removal processes, this research aimed to understand how the different electron acceptors commonly present in biological nutrient removal (BNR) systems can affect the proliferation of SRB. For this purpose, a culture of SRB was enriched in a sequencing batch reactor (approx. 88% of the total bacteria population). Once enriched, the SRB were exposed for 2 h to typical concentrations of electron acceptors like those observed in BNR systems. Their activity was assessed using three different types of electron donors (acetate, propionate and lactate). Oxygen was the most inhibiting electron acceptor regardless the carbon source used. After exposure to oxygen and when feeding acetate, an inactivation time in the sulphate reduction activity was observed for 1.75 h. Once the sulphate reduction activity resumed, only 60% of the original activity was recovered. It is suggested that the proliferation of SRB is most likely to occur in BNR plants with an anaerobic fraction higher than 15% and operating at sludge retention times higher than 20 days (at a temperature of 20 °C). These results can be used to implement strategies to control the growth of sulphate reducers that might compete for organic carbon with phosphate-accumulating organisms.

**Electronic supplementary material:**

The online version of this article (doi:10.1007/s00253-017-8340-3) contains supplementary material, which is available to authorized users.

## Introduction

Sulphate-rich wastewater (containing up to 500 mg SO_4_^2−^/L) can be generated due to (i) discharge of sulphate into the WWTP by industrial effluents (Sears et al. 2004), (ii) seawater and/or groundwater (rich in sulphate) intrusion (van den Brand et al. 2014), (iii) use of sulphate chemicals in drinking water production (e.g. aluminium sulphate, Bratby 2016) and (iv) the use of seawater as secondary quality water (e.g. cooling, toilet flushing) (Lee and Yu 1997).

Heterotrophic dissimilatory sulphate reduction can occur in sulphate-rich waters under anaerobic conditions at COD/SO_4_ ratios higher than 0.66 mg COD/mg SO_4_^2−^ (Liamleam and Annachhatre 2007; Muyzer and Stams 2008). Under anaerobic conditions, sulphate-reducing bacteria (SRB) can compete with anaerobic bacteria for a wide range of carbon sources and electron donors such as glucose, lactate, propionate, acetate, butyrate and ethanol, among others (Muyzer and Stams 2008). The degradation of carbon by sulphate reducers can be divided into two groups: (i) complete degradation to carbon dioxide and (ii) partial degradation to acetate (Liamleam and Annachhatre 2007).

Hydrogen sulphide, the end product of sulphate reduction, is commonly undesired in the treatment of wastewater due to (i) potential corrosion, (ii) interference with other biological/chemical process and (iii) health and safety risks to workers (Londry and Suflita 1999; Okabe et al. 2005). Nevertheless, sulphate reduction can also be beneficial when applied to wastewater treatment as sulphide can be used for (i) heavy metal removal through precipitation (Lewis 2010), (ii) autotrophic denitrification (Kleerebezem and Mendezà 2002; Ginestet et al. 2015) and autotrophic phosphorus removal (Rubio-Rincón et al. 2017) accompanied with reduced BOD requirements for N-removal and P-removal and (iii) reduction of pathogens (Abdeen et al. 2010).

To repress the formation of sulphide, past studies have focused on the development of measures that can inhibit the sulphate-reducing bacteria (SRB) activity. One way to inhibit SRB activity is by avoiding the creation of anaerobic conditions through the addition of oxygen or nitrate (Dilling and Cypionka 1990; Bentzen et al. 1995). Another approach is through the addition of metabolic inhibitors such as molybdate and nitrite (Nemati et al. 2001). Oxygen has shown to be toxic for many anaerobic bacteria, including SRB (Lens and Kuenen 2001). Still, SRB can endure the (short-term or partial) exposure to oxic conditions by (i) the oxygen respiration at the expense of poly-glucose (Kjeldsen et al. 2005; Dolla et al. 2006), (ii) adherence to biofilms where the gradients reduce their exposure to oxygen or other electron acceptors (Lens and Kuenen 2001) and (iii) their potential symbiosis with oxygen-oxidizing organisms (e.g. sulphide-oxidizing bacteria) (van de Ende et al. 1997; Xu et al. 2012, 2014). Moreover, usually if the conditions turn anaerobic again, SRB can recover their activity (Kjeldsen et al. 2005; Nielsen et al. 2008).

Likewise oxygen, nitrate and/or nitrite has been applied to suppress the sulphate reduction process and/or oxidize the sulphide generated back to elemental sulphur or sulphate (Bentzen et al. 1995; Mohanakrishnan et al. 2008, 2009). During the long-term exposure to nitrate, García De Lomas et al. (2006) observed the growth of autotrophic denitrification bacteria capable to use sulphide as an electron donor (*Thiomicrospira*) in an enriched sulphate-reducing biomass. Thus, García De Lomas et al. (2006) suggested that the lower sulphide production observed during the presence of nitrate was not caused by the inhibition of the sulphate reduction process. Instead the later authors suggested that sulphide was used as an electron donor during the denitrification process. During the autotrophic denitrification process, other researchers had observed an accumulation of nitrite in the media (Hubert et al. 2005; Barton and Hamilton 2007). Based on these observations, Barton and Hamilton (2007) suggested that the inhibition of SRB due to nitrate could be related to nitrite formation. Later studies showed that nitrite was able to suppress the reduction process of sulphite (SO_3_^2−^) to sulphide (HS^−^) (Barton and Hamilton 2007; Mohanakrishnan et al. 2008). Interestingly once nitrate and/or nitrite was consumed, the sulphate reduction process resumed reaching an activity similar to that observed before the inhibition occurred (Okabe et al. 2005; Mohanakrishnan et al. 2008). In line with these observations, van den Brand et al. (2015) noticed that while SRB were not active in the aerobic and anoxic zones of the WWTP, their activity resumed after 3 h of exposure to anaerobic conditions; even if minimal, the growth of SRB in WWTPs was possible.

Despite that several studies focused on the inhibition of SRB, caused by their exposure to different electron acceptors (oxygen, nitrate, nitrite), there is a lack of information on the effect of the conditions observed in wastewater treatment plants (WWTPs) on SRB. Therefore, this research aimed to understand how different anoxic and oxic contact times affect the inhibition and activity recovery of SRB under anaerobic conditions. It further discusses which conditions in a WWTP (anaerobic, anoxic and oxic) can be manipulated to either promote or inhibit the growth and activity of sulphate reducers in a WWTP.

## Materials and methods

### Reactor operation

A culture of sulphate reduction bacteria (SRB) was enriched in a double-jacketed Applikon reactor (Delft, The Netherlands) with a working volume of 2.5 L. Activated sludge (500 mL) from WWTP Nieuwe Waterweg (Hoek van Holland, The Netherlands) was used as inoculum. The bioreactor was operated in cycles of 6 h with an effective 5-h anaerobic reaction time, 30-min settling and 30-min effluent removal. In order to ensure the creation of anaerobic conditions (assumed to occur at redox levels lower than −400 mV), nitrogen gas was sparged during the first 20 min of operation and a double water lock was installed and connected to the headspace. The double water lock consisted of one bottle filled with NaOH to capture the sulphide produced and a second bottle with Na_2_SO_3_ + CoCl_2_ to decrease the potential intrusion of oxygen. In order to create good mixing conditions, the biomass was stirred at 500 rpm. During effluent withdrawal, half of the working volume was removed to reach a hydraulic retention time (HRT) of 12 h. The sludge retention time (SRT) was controlled at 15 days by removing 41 mL of mixed liquor sludge at the end of the anaerobic phase. The pH was adjusted at 7.6 ± 0.1 through the addition of 0.4 M HCl and 0.4 M NaOH. Temperature was controlled at 20 ± 1 °C with a water bath. The redox level was monitored continuously online, and it fluctuated between −400 and −480 mV. Sulphate (SO_4_-S), sulphide (H_2_S-S), total suspended solids (TSS) and volatile suspended solids (VSS) were measured twice per week. When no significant changes in these parameters were observed for at least three SRTs (45 days), it was assumed that the system had reached pseudo steady-state conditions.

### Medium

The medium was prepared in two separate bottles of 10 L (one with the COD and the other with the mineral sources, respectively). Each bottle (containing the media solutions) was sterilized at 110 °C for 1 h. The mixed media fed into the reactor contained per litre 93 mg of sodium acetate (43 mg COD), 29 μL of propionic acid (44 mg COD), 216 μL of lactic acid (237 mg COD), 107 mg NH_4_Cl (28 mg NH_4_-N), 112 mg NaH_2_PO_4_·H_2_O (25 mg PO_4_-P), 1.24 g MgSO_4_·7H_2_O (498 mg SO_4_^2−^), 14 mg CaCl_2_·2H_2_O (4 mg Ca^+^), 36 mg KCl (19 mg K^+^), 1 mg yeast extract, 2 mg N-allylthiourea (ATU) and 300 μL of trace element solution prepared according to Smolders et al. (1994).

### Control batch activity tests

Batch activity tests were performed in 500-mL double-jacketed reactors with a working volume of 400 mL. Two hundred millilitres of mixed liquor from the parent reactor (approx. 900 mg VSS/L) was used to conduct each control batch test. After the sludge transfer from the parent to the batch reactor, the waste of sludge of the parent reactor was adjusted to compensate for the withdrawal of biomass. To ensure the creation of anaerobic conditions, nitrogen gas was sparged at the bottom of the batch reactor at a flow rate of 10 L/h during 30 min prior to the start and during the conduction of the control batch activity test. Three carbon sources (acetate, propionate and lactate) were added separately to each set of tests. The batch tests were performed for 6 h following a similar sequence and operation like the parent reactor. The pH and temperature were controlled at 7.6 ± 0.1 and 20 ± 1 °C, respectively. The sludge was continuously stirred at 300 rpm with a magnetic stirring plate. TSS and VSS were measured at the start and end of the test. Acetate, propionate, lactate, sulphide and sulphate were analysed along the execution of the different experiments.

### Batch activity tests executed under the presence of electron acceptors

The residual effects of three electron acceptors (2.7 mg O_2_/L, 15 mg NO_3_-N/L and 10 mg NO_2_-N/L) on the sulphate reduction process using three different electron donors (acetate, propionate and lactate) were assessed separately. In each test, 200 mL of mixed liquor from the parent reactor (approx. 900 mg VSS/L) was transferred to double-jacketed reactors with a working volume of 400 mL each. Two hundred millilitres of the mineral solution (free of organics) used for the enrichment of the SRB culture was added in combination with one of the electron acceptors. Each test lasted for 2 h. Thereafter, the corresponding electron acceptor was removed as described in the following and a reversibility test was conducted to assess the residual inhibition effects on the sulphate reduction process. The reversibility tests were performed following the same conditions like the control batch tests conducted for 6 h.

### Addition and removal of electron acceptors in the inhibitory batch tests

Oxygen was constantly measured and controlled (around 2.7 ± 0.1 mg O_2_/L) by a mixture of compressed air (10 L/h) and nitrogen gas (20 L/h) throughout the corresponding inhibitory batch tests. Once the inhibitory tests concluded (after 2 h), nitrogen gas was sparged during 20 min at 10 L/h until oxygen was no longer detected.

In the beginning of the inhibitory tests, nitrate and nitrite were added from a concentrated stock solution (containing 1 g NO_x_-N/L) to reach a concentration of 15 mg NO_3_-N/L and 10 mg NO_2_-N/L, as corresponded in each test. In order to assess the occurrence of denitrification by endogenous respiration, nitrate and nitrite were measured at the start and end of the tests. At the end of the tests, the nitrate or nitrite compounds were removed by washing the sludge three times. Each washing procedure consisted of a settling phase of 20 min, removal of the supernatant (which comprised approx. 90% of the volume) and the addition of a mineral solution (previously sparged with nitrogen gas) similar to the one used in the control batch tests. After washing the sludge three times, the nitrate/nitrite concentrations were below detection limits (≤0.1 mg NO_x_-N/L). During the washing procedures, nitrogen gas was sparged at the headspace continuously to avoid oxygen intrusion.

### Analyses

Samples for the determination of soluble compounds were filtered through 0.45-μm pore size filters (PDVF). In order to avoid sulphide stripping, the samples used for sulphate and sulphide determination were kept in a 0.5 M NaOH solution (corrected according to the dilution caused by the addition of the NaOH solution). All samples were measured in the subsequent 2 h after the tests concluded. Sulphate was measured by ion chromatography (IC) using a Dionex IonPac AS4A-SC column (Dreieich, Germany). Nitrite, sulphide, TSS and VSS were analysed as described in APHA, AWWA and WEF (2005). Nitrate was measured according to ISO 7890/1 (1986). Acetate and propionate were measured using a Varian 430-GC gas chromatograph (GC) equipped with a split injector (200 °C), a WCOT Fused Silica column (105 °C) and coupled to a FID detector (300 °C). Helium gas was used as carrier gas and 50 μL of butyric acid as internal standard. Lactate was measured in a high-performance liquid chromatography (HPLC) using a Trace 2000 chromatograph (Thermo Electron S.P.A., Milan, Italy).

### Determination of stoichiometric and kinetic parameters of interest

The net carbon consumption per sulphate reduction (mg COD/mg SO_4_^2−^) was calculated based on the total carbon consumption and sulphate reduction observed after 1 h of activity. All kinetic rates were calculated by linear regression as described in Smolders et al. (1995). The rates of interest were as follows:

1. *q*_Ac_: Acetate consumption rate, in mg COD-Ac/g VSS h
2. *q*_Pr_: Propionate consumption rate, in mg COD-Pr/g VSS h
3. *q*_Lac_: Lactate consumption rate, in mg COD-Lac/g VSS h
4. *q*_COD_: Organic carbon consumption rate, in mg COD/g VSS h
5. *q*_SO4_,_Ac_: Sulphate reduction associated with acetate consumption, in mg SO_4_-S/g VSS h
6. *q*_SO4_,_Pc_: Sulphate reduction associated with propionate consumption, in mg SO_4_-S/g VSS h
7. *q*_SO4_,_Lac_: Sulphate reduction associated with lactate consumption, in mg SO_4_-S/g VSS h

### Microbial characterization

Fluorescence in situ hybridization (FISH) analyses were performed according to Amman (1995) to identify the presence of the microbial communities of interest. In order to target all bacteria, equal amounts of EUB 338, EUB338 II and EUB 338 III probes were mixed (EUB MIX) and applied (Nielsen et al. 2009). Most *Desulfovibrionales* and other Bacteria were targeted with the SRB385 probe and most *Desulfobulbus* with the DBB660 probe (Baumgartner et al. 2006; Muyzer and Stams 2008). Vectashield with DAPI was used to amplify the fluorescence and avoid the fading and staining of all organisms (Nielsen et al. 2009). Biomass quantification was performed through image analysis of 20 random pictures taken with an Olympus BX5i microscope and analysed with the software Cell Dimensions 1.5. The standard error of the mean was calculated as described by Oehmen et al. (2010). Denaturing gradient gel electrophoresis (DGGE) was performed as described by Bassin et al. (2011).

## Results

### Performance of the parent reactor

**Fig. 1:**
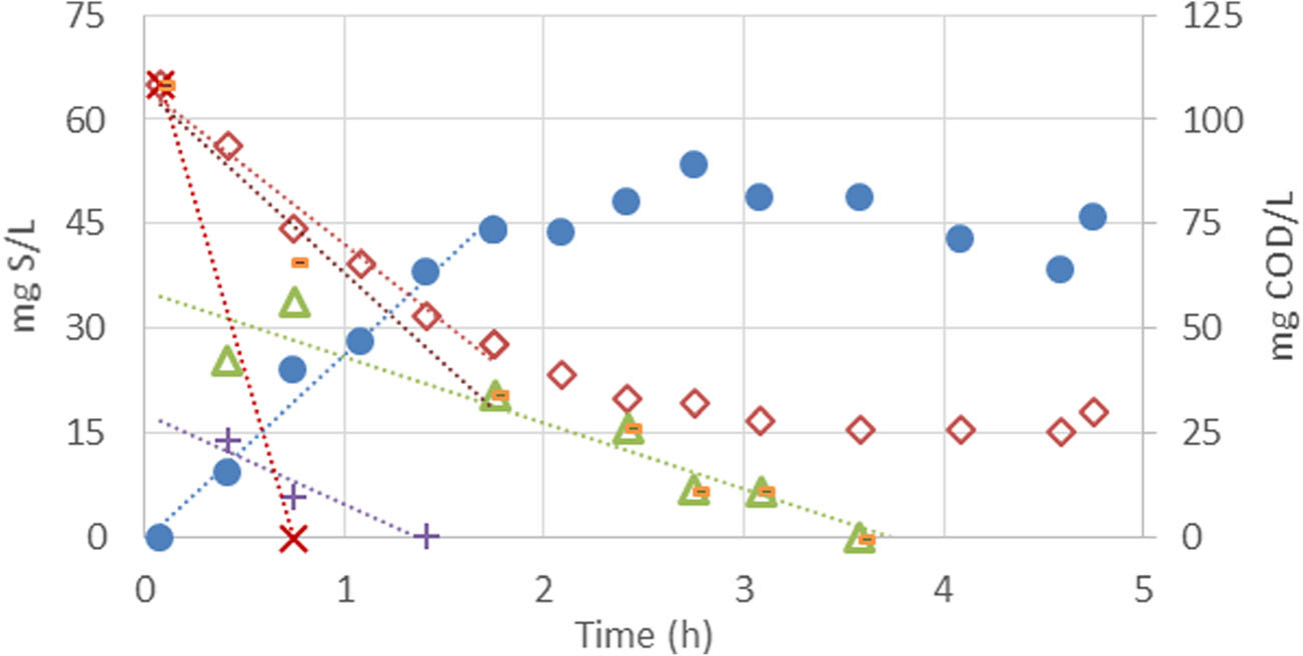
Sulphide (*circles*), sulphate (*diamonds*), acetate (*triangles*), propionate (*plus signs*), lactate (*multiplication signs*) and soluble COD (*dashes*) profiles of the SRB enrichment culture observed during a typical cycle in the parent reactor

In the parent reactor, all carbon sources were consumed in the anaerobic stages. Lactate was consumed at a rate of 185 mg COD-Lac/(g VSS h), while propionate and acetate were consumed at the slower rates of 24.7 mg COD-Pr/(g VSS h) and 20.7 mg COD-Ac/(g VSS h), respectively (Fig. 1). Sulphate was reduced at a rate of 26 mg SO_4_-S/(g VSS h) during the first 2 h of reaction. After this time, it was not possible to observe any considerable change in the sulphate/sulphide concentration in the liquid phase. The COD-conversion/SO_4_-reduction conversion ratio observed in the first 2 h was 0.64 mg COD/mg SO_4_^2−^_._ Considering the fed organic COD (162 mg COD/L), sulphide production (45.8 mg S/L; 91.6 mg COD/L) and biomass formation (36.6 mg VSS/L; 55.6 mg COD/L), the COD balance closed to 91% during the cycle.

**Fig. 2:**
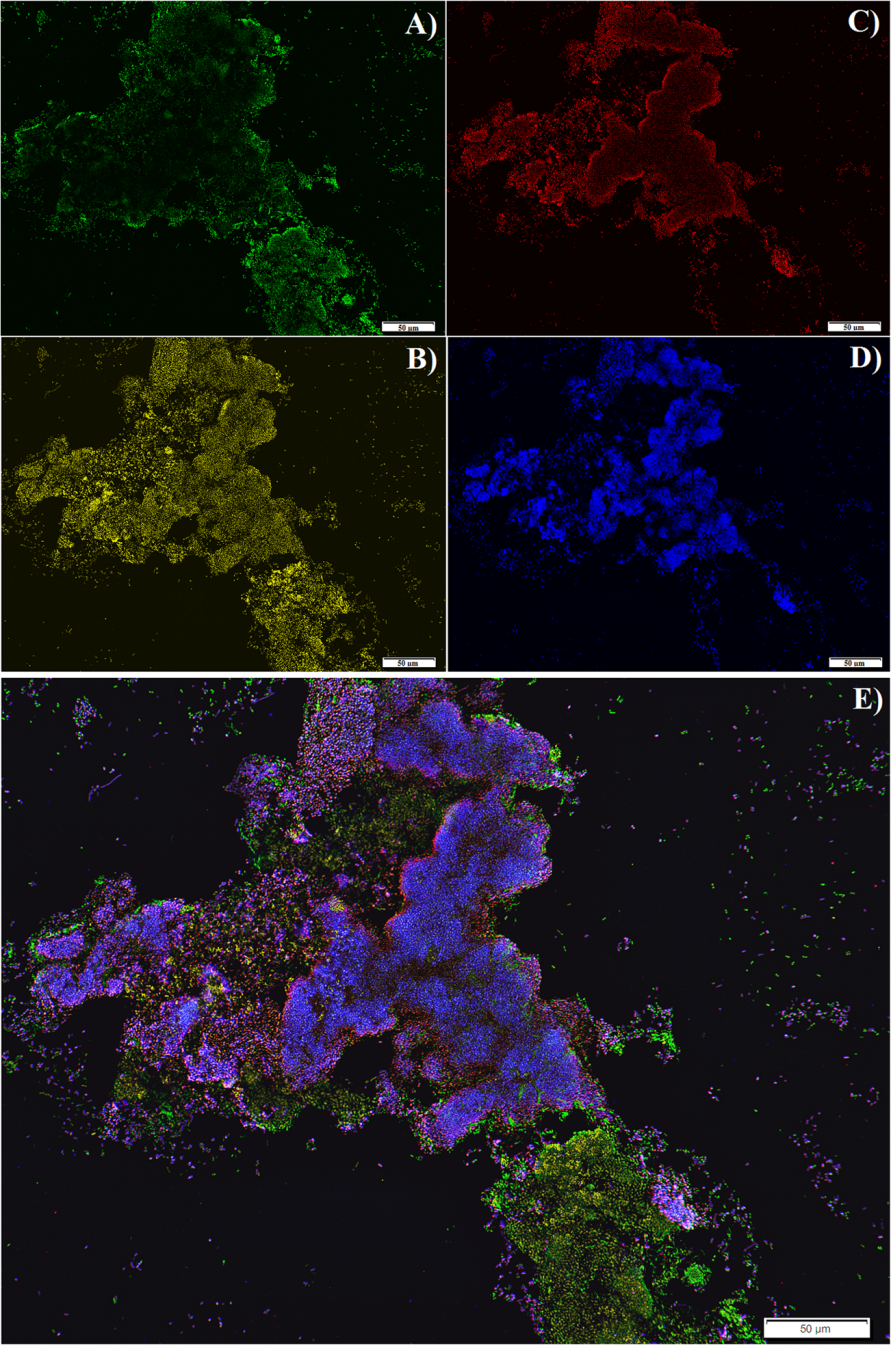
FISH microbial characterization of the biomass present in the parent reactor: **a** DAPI (all living organism), **b** EUB MIX (all bacteria), **c** SRB385 (most sulphate reducers) and **d** DBB660 (*Desulfobulbus*). **e** Overlap of **a**–**d**

**Fig. 3:**
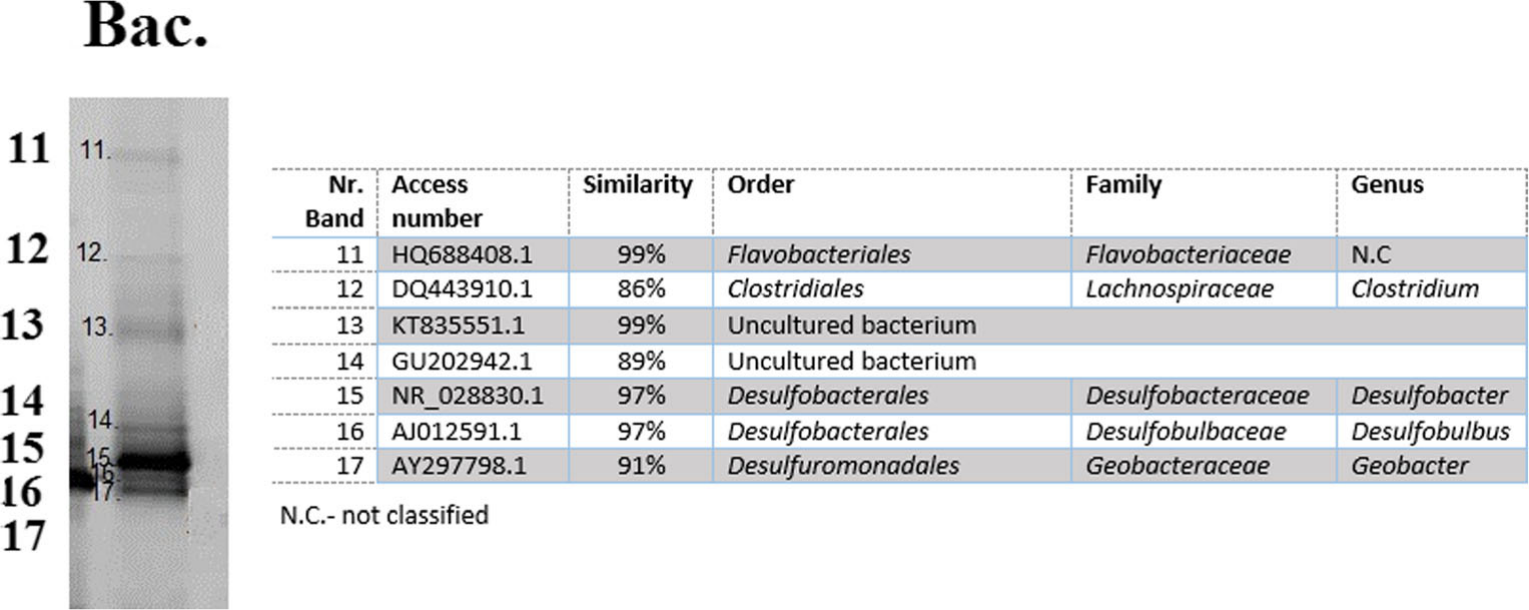
DGGE band pattern and phylogenetic tree of the biomass enriched in the parent reactor

According to the FISH image analyses, the microbial community targeted with the EUB mix probe covered about 79 ± 6% EUB/DAPI (Fig. 2b) of all the cells that reacted with DAPI (Fig. 2a). The bacterial community targeted with the EUB mix probe consisted of 88 ± 4% SRB385/EUB sulphate-reducing bacteria (SRB) (Fig. 2c), from which 96 ± 9% DBB660/SRB385 belonged to the genera *Desulfobulbus* (Fig. 2d)*.* These FISH analyses are in line with the results gathered by denaturing gradient gel electrophoresis (DGGE), which shows a clear presence of *Desulfobulbus* and *Desulfobacter* (Fig. 3).

### Control tests

**Fig. 4:**
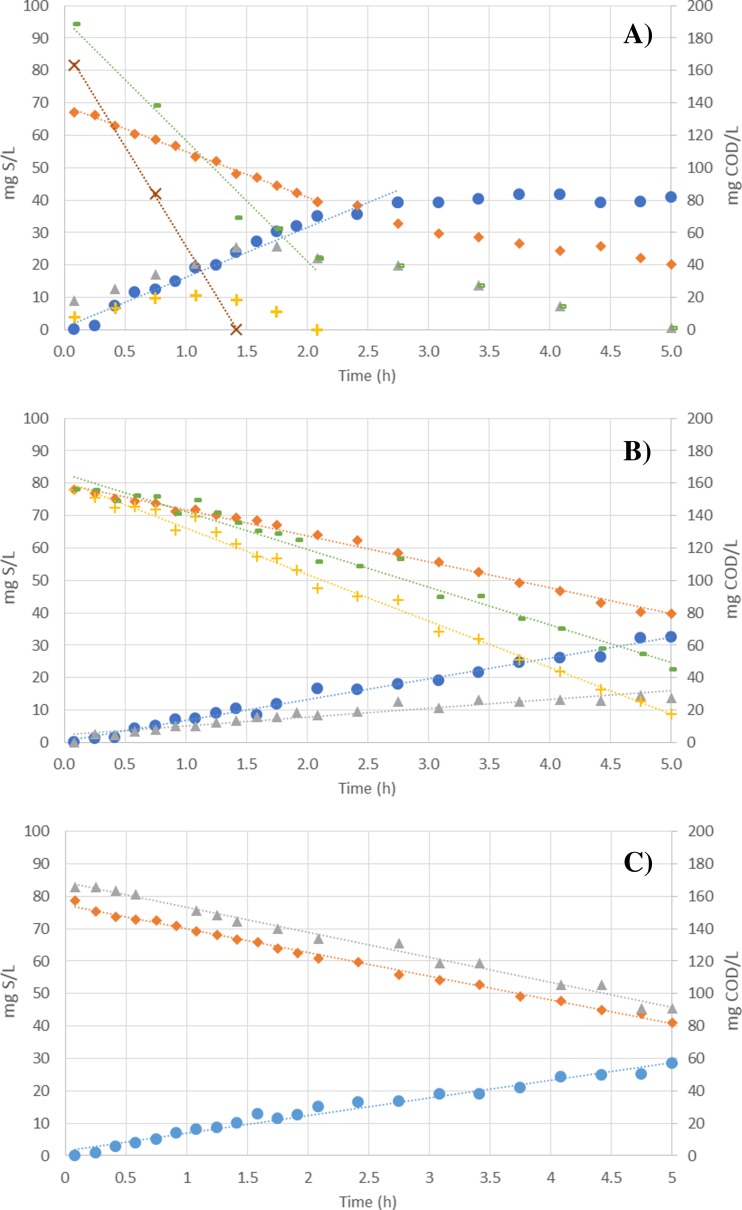
Profiles observed in the control tests showing the concentrations of sulphide (*circles*), sulphate (*diamonds*), acetate (*triangles*), propionate (*plus signs*), lactate (*multiplication signs*), soluble organic COD (*dashes*) profiles in the control test performed with **a** lactate, **b** propionate or **c** acetate as carbon source

The control tests performed with lactate showed a faster COD consumption than the batch test fed with propionate or acetate (1.5 and 4.2 times faster, respectively). Lactate was consumed at a rate of 282.6 mg COD-Lac/g VSS h. During the consumption of lactate, the formation of propionate and acetate was observed. Thus, the overall organic soluble COD consumption was 172 mg COD/(g VSS h) (Fig. 4a). Once lactate was completely removed, propionate and acetate were consumed at the rates of 49.6 mg COD-Pr/(g VSS h) and 35.6 mg COD-Ac/(g VSS h), respectively. In line with the COD consumption, the sulphate reduction associated with lactate consumption occurred at a rate of 32.6 mg SO_4_-S/(g VSS h), which is faster than the sulphate reduction related to acetate consumption of 14.4 mg SO_4_-S/(g VSS h) (Table 1). The overall COD/SO_4_^2−^ consumption ratio was 1.79 mg COD/mg SO_4_^2−^.

**Table 1:** Carbon consumption and sulphate reduction rates observed with the different carbon sources fed to the batch reactor during the first hour of conduction of the control tests and the inhibiting tests executed with oxygen, nitrate and nitrite

		Lag phase	COD/SO_4_	*q*_COD_	*q*_Lac_	*q*_Pr_	*q*_Ac_	*q*_SO4 , Lac_	*q*_SO4 , Pr_	*q*_SO4 , Ac_
		(h)	(mg COD/mg SO_4_)	(mg COD/g VSS.h)	(mg COD/g VSS.h)	(mg COD/g VSS.h)	(mg COD/g VSS.h)	(mg SO_4_-S/g VSS h)	(mg SO_4_-S/g VSS h)	(mg SO_4_-S/g VSS h)
Lactate as carbon source	Control	N.O.	1.79	172.0	282.6	49.6	35.6	32.6	32.2	14.4
	Oxygen test	N.O.	1.58	83.7	154	33.0	48.4	15.1	18.1	11.2
	Nitrate test	N.O.	1.57	113.2	203.6	32.2	23.7	22.1	24.7	12.4
	Nitrite test	N.O.	1.42	94.4	199.4	41.7	19.4	20.6	25.9	10.9
Propionate as carbon source	Control	N.O.	0.97	68.6	N.A.	84.6	N.O.	N.A.	24.0	N.O.
	Oxygen test	0.6	1.07	41.5	N.A.	52.8	N.O.	N.A.	12.9	N.O.
	Nitrate test	0.4	0.90	49.5	N.A.	61.3	N.O.	N.A.	18.3	N.O.
	Nitrite test	0.4	0.97	37.1	N.A.	49.2	N.O.	N.A.	12.7	N.O.
Acetate as carbon source	Control	N.O.	0.70	40.4	N.A.	N.A.	40.4	N.A.	N.A.	19.3
	Oxygen test	1.75	0.83	26.1	N.A.	N.A.	26.1	N.A.	N.A.	10.4
	Nitrate test	0.6	0.90	19.4	N.A.	N.A.	19.4	N.A.	N.A.	7.2
	Nitrite test	0.4	0.83	33.6	N.A.	N.A.	33.6	N.A.	N.A.	13.4

*N.O.* not observed, *N.A.* not applicable

In the control batch test fed with propionate, acetate formation was observed. While propionate was consumed at a rate of 84.6 mg COD-Pr/(g VSS h), acetate was formed at a rate of 16 mg COD-Ac/(g VSS h) and sulphate was reduced at a rate of 24 mg SO_4_-S/(g VSS h) (Fig. 4b). In this experiment, solely 89% of the fed propionate was consumed and acetate removal was not observed. The overall COD/SO_4_^2−^ ratio in the propionate control test was 0.97 mg COD/mg SO_4_^2−^.

The control batch test fed with acetate showed the slowest carbon consumption and sulphate reduction among the three tests executed with the three different carbon sources. Acetate was consumed at a rate of 40.4 mg COD-Ac/(g VSS h) together with 19.3 mg SO_4_-S/(g VSS h) reduction. The COD/SO_4_ consumption ratio observed was the smallest one among the three carbon sources used (of 0.70 mg COD/mg SO_4_^2−^).

### Oxygen inhibition tests

Like in the control batch tests, the organic COD consumption in the lactate test was two times higher than the one observed in the propionate test and 3.2 times higher when compared with the acetate test (Table 1). Compared to the control test, the exposure to oxygen decreased the lactate consumption and the sulphate reduction rates to 154 mg COD-Lac/(g VSS h) and 15 mg SO_4_-S/(g VSS h), respectively. Similarly, the propionate formed was consumed at a slower rate of 33 mg COD-Pr/(g VSS h) coupled to a sulphate reduction rate of 18 mg SO_4_-S/(g VSS h). However, the concentration of acetate formed due to the incomplete lactate oxidation was higher in the oxygen inhibition test than in the control test (61 and 51 mg COD-Ac/L, respectively). Thereafter, the acetate consumption rate was higher than in the control test (48 and 35 mg COD-Ac/(g VSS h), respectively). Interestingly, the sulphate reduction associated with the consumption of the acetate formed was slightly slower than in the control test (of 11 mg SO_4_-S/(g VSS h) and 14 mg SO_4_-S/(g VSS h), respectively).

When propionate was fed as carbon source, the exposure to oxygen caused a 0.6-h longer lag phase in the propionate consumption. After the exposure of the biomass to oxygen, both the propionate consumption and the associated sulphate reduction rates were 48 and 46% slower than the rates observed in the corresponding control test (Table 1). Similar to the control test fed with propionate as carbon source, propionate was not completely consumed and the acetate formed was not consumed either.

In the tests conducted with the addition of acetate as carbon source, no acetate consumption was observed for 1.75 h after the exposure of the biomass to oxygen. Once the activity of the biomass resumed, the acetate consumption rate was 26.1 mg COD-Ac/(g VSS h) and the sulphate reduction rate reached 10.4 mg SO_4_-S/(g VSS h).

### Nitrate inhibition test

During the nitrate inhibition batch tests, the residual activity of SRB after the exposure to nitrate was assessed. The residual activity of SRB after the exposure to nitrate was higher when lactate was fed (Table 1).

The consumption of lactate and sulphate started immediately after the conditions switched from anoxic to anaerobic. Lactate and sulphate were consumed at rates equivalent to 38 and 32% of the rates observed in the control test (Table 1). A similar residual effect was observed in the consumption of the propionate and acetate formed in the test (65 and 66%, respectively). On the other hand, the sulphate reduction associated with the propionate and acetate consumption was higher but still lower than the rate observed in the control (76 and 86%, respectively).

When propionate was used as carbon source, it was not possible to observe any consumption of propionate during the first 0.4 h of the reaction time. After 0.4 h, the propionate consumption rate was 61.3 mg COD-Pr/(g VSS h) coupled with a sulphate reduction rate of 18.3 mg SO_4_-S/(g VSS h). Similar to the oxygen inhibition and control tests, propionate was not completely consumed and no acetate consumption was observed.

A more severe effect was observed on the nitrate inhibitory test fed with acetate. In this experiment, neither acetate consumption nor sulphate reduction was observed in the first 0.6 h. After this period, acetate was consumed at a rate of 19.4 mg COD-Ac/(g VSS h) together with a sulphate reduction rate of 7.2 mg SO_4_-S/(g VSS h).

### Nitrite inhibition test

During the nitrite inhibition test fed with lactate, lactate was consumed at 199.4 mg COD-Lac/(g VSS h) while the propionate and acetate formed were consumed at 41.7 mg COD-Pr/(g VSS h) and 19.4 mg COD-Ac/(g VSS h), respectively (Table 1). Like in the previous batch tests, the sulphate reduction related to the propionate consumption was higher than the sulphate reduction related to lactate consumption (Table 1). On the other hand, the sulphate reduction coupled with the consumption of acetate was around half of that observed with either lactate or propionate (10.9 mg SO_4_-S/(g VSS h)).

In the nitrite tests fed with propionate, it was not possible to observe any activity in the first 0.4 h. Afterwards, a slower propionate consumption of 49.2 mg COD-Pr/(g VSS h) coupled with 12.7 mg SO_4_-S/(g VSS h) was observed. A similar lag phase was observed when acetate was used as carbon source (0.4 h). Nevertheless, the recovery of the acetate consumption rate and the coupled sulphate reduction rate was higher (33.6 mg COD-Ac/(g VSS h) and 13.4 mg SO_4_-S/(g VSS h, respectively)).

## Discussion

### Characterization of biomass performance in the bioreactor

According to the FISH quantification performed during this study, the biomass used for this experiment was mainly SRB (approx. 88% SRB385/EUB) and more specifically *Desulfobulbus* (approx. 96% DBB660/SRB 385). This is in line with the calculations for the observed biomass growth showed in supplementary material A, when compared with the observed growth reported in similar systems. This research aims to understand the residual activity of SRB after their exposure to different electron acceptors. Therefore, as previous research has pointed out, the effects of toxic and inhibitory compounds on SRB are dependent on the carbon source consumed (Maillacheruvu and Parkin 1996). Thus, this study assessed the effects of the consumption of each carbon source on the sulphate reduction activity. As observed in Fig. 1, part of the lactate fed (26%) was fermented to propionate and acetate. According to Oyekola et al. (2012), the half-saturation constant of lactate-oxidizing organisms is 0.13 g COD-Lac/L, whereas for lactate fermenters, it is 3.56 g COD-Lac/L. Thus, the lower concentration of lactate added in this study (0.23 g COD-Lac/L) was supposed to be beneficial for the oxidation process over the fermentation process of lactate, which was not the case. It is estimated that 49% of the total lactate was incompletely oxidized with sulphate to acetate (supplementary material B). This is in line with the results presented by Dar et al. (2008) who suggested that incomplete oxidizing SRB outcompete complete oxidizers. According to the Gibbs free energy presented in Table 2, SRB can generate twice as much energy during the incomplete oxidation of lactate compared with its complete oxidation (−160.3 and −84.9 kJ/mol S, respectively). This might explain why the lactate was largely incompletely oxidized into acetate by SRB.

**Table 2:** Possible sulphate reduction reactions in an enrich SRB bioreactor

Equation	∆*G*_o_^’a^ (kJ/reaction)	∆*G*_o_^’^ (kJ/mol S)
(2) C_2_H_3_O_2_^−^ + SO_4_^2−^ → HS^−^ + 2HCO_3_^−^	−47.3	−47.3
(3) 4C_3_H_5_O_2_^−^ + 3SO_4_^2−^ → 3HS^−^ + 4HCO_3_^−^ + 4C_2_H_3_O_2_^−^ + H^+^	−151.3	−50.4
(4) 4C_3_H_5_O_2_^−^ + 7SO_4_^2−^ → 7HS^−^ + 12HCO_3_^−^ + H^+^	−340.5	−48.6
(5) 3C_3_H_5_O_3_^−^ → C_2_H_3_O_2_^−^ + 2C_3_H_5_O_2_^−^ + CO_2_ + H_2_O	−170.0	N.A.
(6) 2C_3_H_5_O_3_^−^ + SO_4_^2−^ → HS^−^ + 2HCO_3_^−^ + 2C_2_H_3_O_2_^−^ + H^+^	−160.3	−160.3
(7) 2C_3_H_5_O_3_^−^ + 3SO_4_^2−^ → 3HS^−^ + 6HCO_3_^−^ + H^+^	−254.9	−84.9

^a^∆*G*_o_^’^ values taken from Thauer et al. (1977)

### Effects of aerobic exposure time on SRB

After the biomass was exposed to oxygen, the residual sulphate activity was similar in all tests independently of the carbon source used (Table 1). This is in agreement with the observations of Cypionka (1994) who concluded that some species of sulphate reducers (e.g. *Desulfovibrio*, *Desulfobulbus*) were capable to survive a continuous exposure to oxygen. As observed in Figs. 2 and 3, at least one of these species was present in the bioreactor.

According to Kjeldsen et al. (2004), the decrease in the sulphate reduction activity observed after the exposure to oxygen could be partially caused by the inhibition of fermentative anaerobic bacteria, which can generate substrate for SRB. However, in this case, SRB were capable to directly use lactate. Interestingly, the recovery in the organic carbon uptake rate was somewhat lower with lactate (48%) than with propionate (60%) or acetate (65%), whereas the sulphate reduction associated with the corresponding carbon consumption was not considerably different (approx. 53 ± 2%). Thus, it might be that the anaerobic bacteria which consumes lactate were more severely affected than SRB by the exposure to oxygen.

Despite similar inhibition activities during the sulphate reduction (approx. 53%), the inactivation time (lag phase) was different according to the carbon source feed. Whereas the lag phase observed with acetate was of 1.75 h, it was inexistent with lactate. This suggests that the exposure to oxygen should not hinder the growth of SRB able to use lactate. This is in line with the studies of Lens and Poorter (1995) who identified that SRB capable to use lactate as carbon source were present in aerobic WWTPs.

Following a similar approach as the one used in the cycle of the parent reactor and using the equations displayed in Table 2, it is possible to calculate the use of carbon source related to sulphate consumption (supplementary material C). This approach shows that the relative fermentation of lactate increased from 18 to up to 35% once the biomass was exposed to oxygen. On the other hand, the relative percentage of lactate oxidation related to sulphate consumption remained similar in both cases (approx. 27%), whereas the lactate oxidized to carbon dioxide decreased from 56 to 37% in the control batch and oxygen stress tests, respectively (supplementary information C). As it is possible to observe on Table 2, the incomplete oxidation of lactate generates twice as much energy per mole of sulphate when compared to the complete oxidation of lactate. Thus, the incomplete oxidation of lactate might provide SRB extra energy needed for the detoxification process. This is in line with Maillacheruvu and Parkin (1996), who suggested that the inhibitory and toxic compound effects on SRB were dependent on the carbon source consumed.

As the SRB that oxidize acetate or propionate were more inhibited by oxygen and because the fraction of lactate fermented increased, the exposure of biomass to oxygen could result in the accumulation of volatile fatty acids (VFAs) in anaerobic selectors. Therefore, the accumulation of VFA could result beneficial for other microbial processes such as denitrification or the biological removal of phosphorus. Nevertheless, the sulphide produced by SRB could hinder the anaerobic and more severely the aerobic metabolism of *Candidatus Accumulibacter phosphatis*, which are the main organisms responsible for the biological removal of phosphorus (Rubio-Rincón et al. 2016).

### Effects of anoxic exposure time on SRB

Past research had suggested the use of nitrate or nitrite as inhibitory compounds to suppress the sulphate reduction activity (Bentzen et al. 1995; Greene et al. 2003; García De Lomas et al. 2006). It is assumed that nitrite (and not nitrate) is the compound that actually causes the inhibition of the dissimilatory sulphate reduction pathway from sulphite onwards (Hubert et al. 2005; Okabe et al. 2005; Barton and Hamilton 2007). However, in this research, sulphide was generated as a product of the sulphate reduction process in the biomass previously exposed to nitrate or nitrite. Possibly, the latter occurred because nitrate and nitrite were not present during the sulphate reduction process. This confirms that the effect of nitrate or nitrite in the reduction of sulphate is reversible, as previously reported (Greene et al. 2003; Kjeldsen et al. 2004; Mohanakrishnan et al. 2008).

In this study, nitrate showed to affect more severely the recovery of the sulphate reduction activity on acetate-consuming SRB than in lactate/propionate-SRB (36 and 76%, respectively). This is in agreement with Maillacheruvu et al. (1993) who observed a higher tolerance to toxic compounds of SRB able to oxidize lactate or glucose compared to SRB that oxidize acetate or propionate. This, as previously explained, could be due to that the higher energy is generated per mole of sulphate by the incomplete oxidation of lactate.

Moreover, the inactivation period of the sulphate reduction activity when the system was fed with acetate was 0.6 and 0.4 h when the biomass was previously exposed either to nitrate or nitrite, respectively. In contrast, the sulphate reduction activity resumed immediately when the system was fed with lactate, suggesting that fermentative and lactate SRB were active. Such inactivation time suggests that acetate cannot be immediately consumed. Kjeldsen et al. (2004) suggested that the lag phase or period of inactivation was related to the different microbial communities. In their experiments, they suggest that the lag phase was caused by the inhibition of fermentative bacteria. Thus, the differences observed in these experiments could be due to the presence of different SRB with different capacities to tolerate the presence of electron acceptor. Greene et al. (2003) suggested that the nitrate reductase enzyme (Nrf) was widely distributed among SRB and could be used for detoxification processes. In that case, the ability of different SRB to express this enzyme (or the use of different carbon sources) might result in the different periods of inactivation observed in this research.

### Possible proliferation of SRB in WWTP

**Fig. 5:**
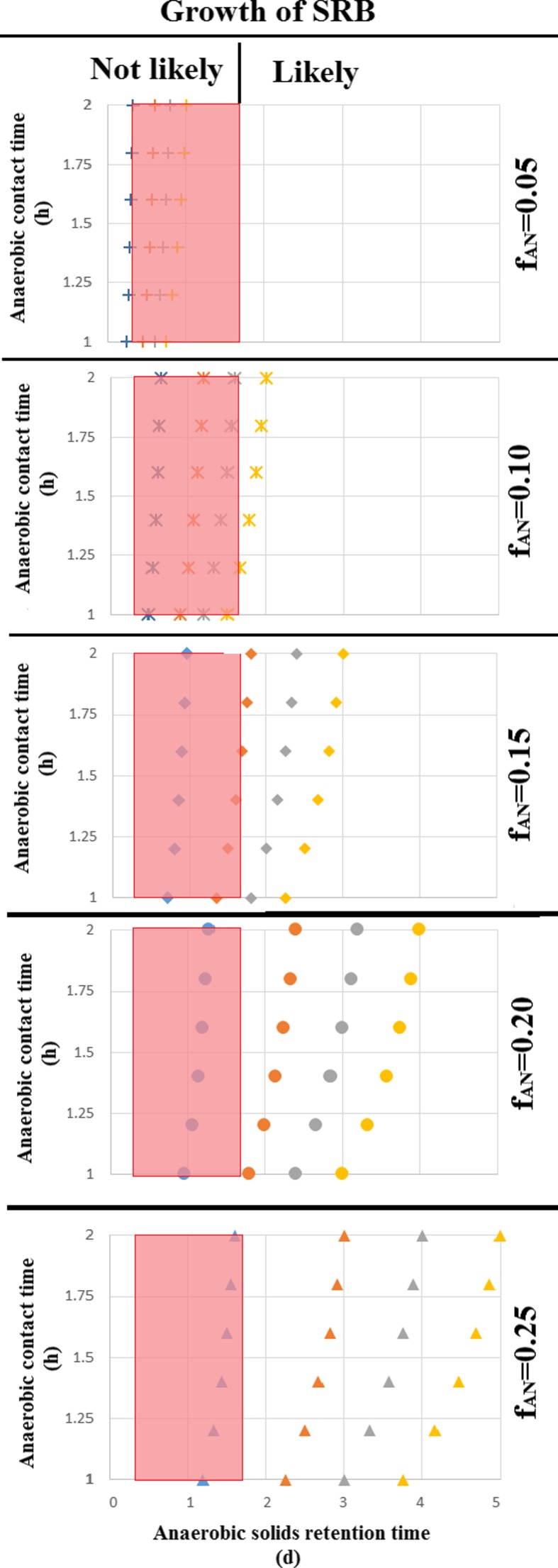
Likelihood of lactate sulphate reducers growing in a BNR plant at different anaerobic contact times (1 to 2 h), anaerobic fractions (5 to 20%) and 8 days (*blue*), 15 days (*orange*), 20 days (*grey*) or 25 days (*yellow*) SRT at 20 C. Considering that the sludge is flocculent, i.e., there is no limitation of substrate and considering a minimum lag phase of 0.4 h for the SRB as observed in this study (Colour figure online)

The inactivation time observed in these past experiments suggests that the anaerobic time in a WWTP for SRB is shortened with the length of their inactivation time. The net anaerobic contact time should be long enough to allow the growth of SRB, in order allow SRB to proliferate in the WWTP. This so-called active anaerobic time where SRB could potentially grow would depend on (i) anaerobic fraction of the WWTP (*f*_an_) which can vary from 0.05 to 0.25 for enhanced biological phosphorus removal (EBPR), (ii) the anaerobic contact time which can vary from 1 to 2 h (Henze et al. 2008) and (iii) the overall applied SRT of the WWTP. Figure 5 shows the theoretical potential for SRB growth as a function of the net anaerobic contact time at different SRTs and anaerobic fractions of the WWTP, when it is considered that (i) there is no substrate limitation, (ii) an inactivation time for SRB of 0.4 h (nitrite inhibitory test) and (iii) the minimum SRT calculated based on the growth rate reported for lactate oxidizers SRB by Oyekola et al. (2012) and Traore et al. (1982) (supplementary material D). According to these calculations, SRB are likely to grow in WWTP with a *f*_an_ higher than 15% in combination with an SRT higher than 20 days. These numbers are for 20 °C, at higher temperatures, or when there is substantial augmentation of SRB from the sewer system, SRB can proliferate at shorter SRTs. This indicates that WWTP that aims to promote the biological removal of phosphorus more likely will develop SRB.

Recent research has shown that sulphide (produced during the sulphate reduction process) can be used as energy source for autotrophic biological phosphorus removal (Rubio-Rincon et al. 2017) and for autotrophic denitrification (Reyes-Avila et al. 2004; García De Lomas et al. 2006; Wang et al. 2009). This study assessed the separate residual effect of electron acceptors on the sulphate-reducing process. Nevertheless, according to the WWTP configuration, the sludge will be exposed to different anoxic and oxic contact times. Thus, the combined effect of anoxic and oxic contact times on SRB should be further studied.

## Electronic supplementary material

ESM 1 (PDF 665 kb)
