# Supplementary material for: Effects of electron acceptors on sulphate reduction activity in activated sludge processes
Source: Appl Microbiol Biotechnol. 2017 May 25;101(15):6229–40. doi: 10.1007/s00253-017-8340-3 (PMC5522498; doi:10.1007/s00253-017-8340-3)
Supplement: Supplementary file 1 — (PDF 665 kb) [file 253_2017_8340_MOESM1_ESM.pdf]

Applied Microbiology and Biotechnology

# Effects of electron acceptors on sulphate reduction activity in activated sludge processes

**Francisco Rubio-Rincón [a,b*], Carlos Lopez-Vazquez [a], Laurens Welles [a, b], Tessa van den Brand [c], Ben Abbas[b], Mark van Loosdrecht [b], Damir Brdjanovic [a, b]**

[a] Sanitary Engineering Chair Group. Department of Environmental Engineering and Water Technology, UNESCO-IHE Institute for Water Education, Westvest 7, 2611AX Delft, The Netherlands. (E-mail: f.rubiorincon@unesco-ihe.org; c.lopezvazquez@unesco-ihe.org; l.welles@unesco-ihe.org; b.brdjanovic@unesco-ihe.org).

[b]. Department of Biotechnology, Delft University of Technology, Van der Maasweg 9, 2629 HZ Delft, The Netherlands. (E-mail: F.J.RubioRincon@tudelft.nl; m.c.m.vanloosdrecht@tudelft.nl; d.brdjanovic@tudelft.nl ).

[c]. KWR Watercycle Research Institute, Groningenhaven 7, 3433 PE Nieuwegein, The Netherlands ( E-mail: tessa.van.den.brand@kwrwater.nl).

* franciscojrubiorincon@gmail.com ; Tel: 0031 (0) 15 215 1715

## Supplementary material A

Based on the net COD and $SO_4^{2-}$ transformations (108 mg COD/L and 45 mg S/L; Figure 1) in the parent reactor, the COD/$SO_4^{2-}$ conversion ratio was around 0.80 mg COD/mg $SO_4^{2-}$. This ratio is higher than the ratio of 0.64 mg COD/mg $SO_4^{2-}$ calculated based on the conversion in the first two hours of reaction time which is close to the theoretical minimal COD consumption per sulphate reduced ratio (Choi et al., 1991). If the theoretical ratio of 0.66 mg COD/mg $SO_4^{2-}$ for catabolism is compared to the measured values, is possible to assess that 84% of the COD could be consumed by SRB for the catabolic process (step 1 "Percentage of COD consumed by SRB"). Based on this COD consumption and the quantification of the SRB using FISH analysis ( approx. 88% SRB/EUB), it is possible to estimate the observed biomass yield using Equation 1 (Henze et al., 2008). In this case, the observed biomass yield of SRB would be between 0.077 mg VSS/mg COD and 0.091 mg VSS/mg COD considering that either all or only 83% of the organic COD fed was consumed by SRB (step 2 "Calculation of observed biomass yield"). The calculated observed growth is in line with the 36 mg VSS removed per cycle to control the SRT in the system at 15 d (25 mg VSS and 30 mg VSS, according to the observed growth of 0.077 mg VSS/mg COD and 0.093 mg VSS/mg COD, respectively). Furthermore, these values are in line with the ones reported for other SRB cultures enriched in similar conditions than the ones applied in this study (pH 7.6, 20°C, using acetate and propionate as carbon source) (Lens et al., 2002; van den Brand et al., 2014). Therefore, based on the microbial characterization and conversion ratios observed in the parent reactor, sulphate reducing bacteria seems to have been the dominant organisms present in the parent reactor (Figures 1, 2 and 3).

$$Y_{obs} = \frac{MxVss}{Qi \cdot Sbi \cdot SRT} = \frac{VSS \cdot V}{Qi \cdot Sbi \cdot SRT} \qquad \text{Equation 1}$$

Where:

| | | |
|---|---|---|
| V: | volume of reactor | L |
| VSS: | volatile suspended solids | mg VSS/L |
| Qi: | influent flow | L/d |
| Sbi: | Soluble biodegradable COD | mg COD/L |
| SRT: | Sludge retention time | d |
| MxVSS: | Mass of volatile suspended solids | mg VSS |

### 1. Percentage of COD consumed by SRB

**Data**

Observed 0.80 mg COD/mg $SO_4^{2-}$ratio in the parent reactor

Theoretical ratio of 0.66 mg COD/mg $SO_4^{2-}$ according to Choi et al. (1991).

**Calculation**

%of COD consumed by SRB= 0.66*100/0.80=82.5% (approx. 83%)

### 2. Calculation of observed biomass yield

**Assumptions**

COD

- All COD is consumed by SRB
- 83 % of the COD is consumed by SRB

**Equation**

$$Y_{obs} = \frac{MxVss}{Qi \cdot Sbi \cdot SRT} = \frac{VSS \cdot V}{Qi \cdot Sbi \cdot SRT} \quad \text{Equation 1}$$

Where:

| | | |
|---|---|---|
| V: | volume of reactor | L |
| VSS: | volatile suspended solids | mg VSS/L |
| Qi: | influent flow | L/d |
| Sbi: | Soluble biodegradable COD | mmol COD/L |
| SRT: | Sludge retention time | d |
| MxVSS: | Mass of volatile suspended solids | mg VSS |

**Data**

V: 2.5L
VSS: 900 mg VSS/L
Qi: 6L/d (1.25 L/cycle, 4 cycles per day)
Sbi: 323 mg COD/L (based on feed concentration of lactate, propionate and acetate)

**Calculations**

- All COD is consumed by SRB

$$Y_{obs} = \frac{VSS \cdot V}{Qi \cdot Sbi \cdot SRT} = \frac{\frac{900 mgVSS}{L} \cdot 2.5L}{\frac{6L}{d} \cdot 323\ mg\ COD/L \cdot 15d} = \frac{0.077\ mgVSS}{mg\ COD}$$

- 84 % of the COD is consumed by SRB

$$Y_{obs} = \frac{VSS \cdot V}{Qi \cdot Sbi \cdot SRT} = \frac{\frac{900 mgVSS}{L} \cdot 2.5L}{\frac{6L}{d} \cdot 323\ mg\ COD/L \cdot 15d \cdot 0.83} = \frac{0.093\ mgVSS}{mg\ COD}$$

## Supplementary material B

### 3. Pathway of oxidation of lactate by SRB in the parent reactor

**Assumptions**

- The reactions occur in an order according to its energy generation per mol of sulphur
- Propionate not acetate are consumed while lactate is being consumed.

**Equation**

| | $\Delta G_o$'[a] (KJ/reaction) | $\Delta G_o$' (KJ/mol S) |
|---|---|---|
| (2) $C_2H_3O_2^- + SO_4^{2-} \rightarrow HS^- + 2HCO_3^-$ | -47.3 | -47.3 |
| (3) $4C_3H_5O_2^- + 3SO_4^{2-} \rightarrow 3HS^- + 4HCO_3^- + 4C_2H_3O_2^- + H^+$ | -151.3 | -50.4 |

| | | | |
|---|---|---|---|
| (4) | $4C_3H_5O_2^- + 7SO_4^{2-} \rightarrow 7HS^- + 12HCO_3^- + H^+$ | -340.5 | -48.6 |
| (5) | $3C_3H_5O_3^- \rightarrow C_2H_3O_2^- + 2C_3H_5O_2^- + CO_2 + H_2O$ | -170.0 | N.A. |
| (6) | $2C_3H_5O_3^- + SO_4^{2-} \rightarrow HS^- + 2HCO_3^- + 2C_2H_3O_2^- + H^+$ | -160.3 | -160.3 |
| (7) | $2C_3H_5O_3^{--} + 3SO_4^{2-} \rightarrow 3HS^- + 6HCO_3^- + H^+$ | -254.9 | -84.9 |

**Data**

Lactate consume: 108.5 mgCOD-Lac (1.13 mmol Lac)

Propionate produced: 23 mg COD-Pr (0.20 mmol Prop)

Acetate produced: 42 mg COD-Ac (0.66 mmol Ac)

**Calculations**

- **Lactate fermented (Equation 5)**

$$0.30\ C_3H_5O_3^- \rightarrow 0.10\ C_2H_3O_2^- + 0.20\ C_3H_5O_2^- + 0.10\ CO_2 + 0.10\ H_2O$$

- **Incomplete oxidation of lactate by SRB (Equation 6)**

$$0.56\ C_3H_5O_3^- + 0.28\ SO_4^{2-} \rightarrow 0.28\ HS^- + 0.56\ HCO_3^- + 0.56\ C_2H_3O_2^- + 0.28\ H^+$$

**Results**

Lactate (fermented) = 0.30 mml Lac (26%)

Lactate (Incomplete oxidation)= 0.56 mmol Lac (49%)

Lactate (Complete oxidation)= 1.13-0.30-0.56= 0.27 mmol Lac (24%)

## Supplementary material C

**4. Pathway of oxidation of lactate by SRB in control and oxygen inhibitory batch test**

**Assumptions**

- The reactions occur in an order according to its energy generation per mol of sulphur
- Propionate not acetate are consumed while lactate is being consumed.

**Equation**

| | $\Delta G_o'^{a}$ (KJ/reaction) | $\Delta G_o'$ (KJ/mol S) |
|---|---|---|
| (2) $C_2H_3O_2^{-}+SO_4^{2-}\rightarrow HS^{-}+2HCO_3^{-}$ | -47.3 | -47.3 |
| (3) $4C_3H_5O_2^{-}+3SO_4^{2-}\rightarrow 3HS^{-}+4HCO_3^{-}+4C_2H_3O_2^{-}+H^{+}$ | -151.3 | -50.4 |
| (4) $4C_3H_5O_2^{-}+7SO_4^{2-}\rightarrow 7HS^{-}+12HCO_3^{-}+H^{+}$ | -340.5 | -48.6 |
| (5) $3C_3H_5O_3^{-}\rightarrow C_2H_3O_2^{-}+2C_3H_5O_2^{-}+CO_2+H_2O$ | -170.0 | N.A. |
| (6) $2C_3H_5O_3^{-}+SO_4^{2-}\rightarrow HS^{-}+2HCO_3^{-}+2C_2H_3O_2^{-}+H^{+}$ | -160.3 | -160.3 |
| (7) $2C_3H_5O_3^{--}+3SO_4^{2-}\rightarrow 3HS^{-}+6HCO_3^{-}+H^{+}$ | -254.9 | -84.9 |

**Data**

| **Compound** | **Control batch test** | | **Oxygen inhibitory batch test** | |
|---|---|---|---|---|
| **Units** | **mg COD** | **mmol (compound)** | **mg COD** | **mmol (compound)** |
| Lactate | 79 | 0.82 | 119 | 1.24 |
| Propionate | 12 | 0.10 | 33 | 0.29 |
| Acetate | 16.5 | 0.26 | 31.5 | 0.49 |

**Calculations**

**Control batch test**

- **Lactate fermented (Equation 5)**

$$0.15\ C_3H_5O_3^{-}\rightarrow 0.05\ C_2H_3O_2^{-}+0.10\ C_3H_5O_2^{-}+0.05\ CO_2+0.05\ H_2O$$

- **Incomplete oxidation of lactate by SRB (Equation 6)**

$$0.21\ C_3H_5O_3^{-}+0.10\ SO_4^{2-}\rightarrow 0.10\ HS^{-}+0.21\ HCO_3^{-}+0.21\ C_2H_3O_2^{-}+0.10\ H^{+}$$

**Oxygen inhibitory batch test**

- **Lactate fermented (Equation 5)**

$$0.43\ C_3H_5O_3^{-}\rightarrow 0.14\ C_2H_3O_2^{-}+0.29\ C_3H_5O_2^{-}+0.14\ CO_2+0.14\ H_2O$$

- **Incomplete oxidation of lactate by SRB (Equation 6)**

$$0.34\ C_3H_5O_3^{-}+0.17\ SO_4^{2-}\rightarrow 0.17\ HS^{-}+0.34\ HCO_3^{-}+0.17\ C_2H_3O_2^{-}+0.17\ H^{+}$$

**Results**

| Lactate | Control batch test | | Oxygen inhibitory batch test | |
|---|---|---|---|---|
| **Units** | **mmol Lac** | **%** | **mmol Lac** | **%** |
| Total | 0.82 | 100 % | 1.24 | 100 % |
| Fermented | 0.15 | 18 % | 0.43 | 35 % |
| Incomplete Digested | 0.21 | 25 % | 0.34 | 28 % |
| Complete oxidation | 0.46 | 56 % | 0.46 | 37 % |

## Supplementary material D

**Calculation of minimal SRT necessary for SRB growth**

**Assumptions**

- The sludge is flocculent
- No substrate limitation
- A lag phase of 0.4 h (nitrite inhibitory test)
- Minimum SRT calculated based on the growth rate reported for lactate oxidizers SRB by Oyekola et al. (2012) and Traore et al. (1982) of approximately 1.8 d.

**Equations**

$$SRT_{AN} = \frac{HRT_{AN} - Lag\ phase}{\frac{HRT_{AN}}{f_{AN}}} * SRT$$

Where:

| | | |
|---|---|---|
| $SRT_{AN}$: | Sludge retention time of the anaerobic tank | d |
| SRT: | Sludge retention time of the WWTP | d |
| $HRT_{AN}$: | Hydraulic retention time of the anaerobic tank | h |
| $f_{AN}$: | Anaerobic fraction of the WWTP | fraction |
| lag phase: | Inactivation time of SRB | h |

**Example calculation**

**Data**

| | |
|---|---|
| SRT: | 20 d |
| $HRT_{AN}$: | 1.5 h |
| $f_{AN}$: | 15 % |
| lag phase: | 0.4 h |

$$SRT_{AN} = \frac{1.5\ h - 0.4\ h}{\frac{1.5\ h}{0.15}} * 20 = 2.2\ days$$

**Results**

At an SRT of 20d, $f_{AN}$ of 15%, 1.5 h anaerobic contact time and 0.4 h of lag phase the anaerobic tank would provide an SRT of 2.2 days for the growth of SRB, which is higher to the minimum of 1.8 days necessary for growth reported by Oyekola et al. (2012) and Traore et al. (1982). Thus under these conditions the proliferation of SRB in the WWTP is likely to happen.
